# Cyclodextrin-Based Delivery Systems and Hydroxycinnamic Acids: Interactions and Effects on Crucial Parameters Influencing Oral Bioavailability—A Review

**DOI:** 10.3390/pharmaceutics14112530

**Published:** 2022-11-20

**Authors:** Kleyton Santos Veras, Flávia Nathiely Silveira Fachel, Valquiria Linck Bassani, Helder Ferreira Teixeira, Letícia Scherer Koester

**Affiliations:** Programa de Pós-Graduação em Ciências Farmacêuticas, Faculdade de Farmácia, Universidade Federal do Rio Grande do Sul, Avenida Ipiranga 2752, Porto Alegre 90610-000, Rio Grande do Sul, Brazil

**Keywords:** phenolic acids, cyclodextrins, binding constant, cyclodextrin complexes, aqueous solubility, stability

## Abstract

Hydroxycinnamic acids (HCAs) are a subclass of phenolic acids presenting caffeic acid (CA), chlorogenic acid (CGA), coumaric acid (COA) isomers, ferulic acid (FA), and rosmarinic acid (RA) as the major representants, being broadly distributed into vegetal species and showing a range of biological potentials. Due to the low oral bioavailability of the HCAs, the development of delivery systems to promote better administration by the oral route is demanding. Among the systems, cyclodextrin (CD)-based delivery systems emerge as an important technology to solve this issue. Regarding these aspects, in this review, CD-based delivery systems containing HCAs are displayed, described, and discussed concerning the degree of interaction and their effects on crucial parameters that affect the oral bioavailability of HCAs.

## 1. Introduction

**Figure 1:**
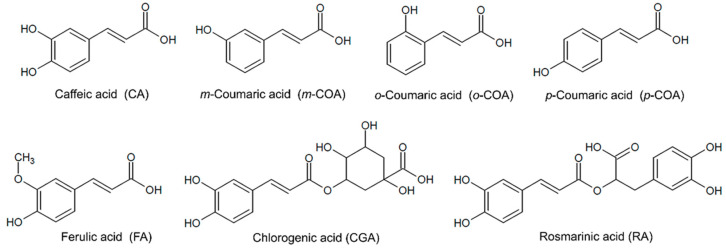
Chemical structures of HCAs.

Phenolic acids are a class of phenolic compound that present in their chemical structure a carboxylic acid and could be subdivided in benzoic and hydroxycinnamic acids (HCAs). HCAs are compounds derived from cinnamic acid, presenting caffeic acid (CA), chlorogenic acid (CGA), coumaric acid (COA) isomers, ferulic acid (FA), and rosmarinic acid (RA) as the major representants (Figure 1) [1,2].

HCAs are found in food sources such as artichokes, black and white beans, broccoli, carrot, cauliflower, coffee, eggplant, garlic, lettuce, potato, and white wine, among others, and their intake has been associated with several health benefits [2,3,4]. In fact, the pharmacological evaluations of the isolated HCAs have shown activity against brain dysfunctions, diabetes, inflammation, hypertension, kidney injury, liver injury, obesity, and oxidative stress [4,5].

However, despite the great therapeutic potential of HCAs, pharmacokinetic studies have demonstrated for these compounds a low oral bioavailability, which could decrease its pharmacological activities [2,6]. In face of the growing interest in the HCAs, lipid-core nanocapsules [7], self-microemulsifying [8,9], nanoparticles [10,11,12], and phospholipid complexes [13] delivery systems, as well as metabolism inhibitors [14,15], have been applied to overcome the low bioavailability of HCAs.

Cyclodextrin (CD)-based delivery systems represent a promising technological strategy widely employed to increase the oral bioavailability of drugs and phytochemical compounds, including HCAs [16,17,18]. In this regard, this review aims to present and discuss the CD-based delivery systems containing HCAs with a focus on interaction features and the effects on aspects responsible for regulating oral bioavailability of HCAs.

Cyclodextrins (CDs) are cyclic oligosaccharides formed by (α-1,4-)-linked D-glucopyranose units, presenting a truncated cone or torus shape, with a lipophilic cavity and hydrophilic outer surface [19]. The lipophilic cavity is able to include drug moieties by noncovalent linkage forming inclusion complexes [20]. Other types of interactions between CDs and drugs have been reported in the literature, such as noninclusion complexes and water-soluble aggregates [21]. These interactions are responsible for modifying the drugs’ solubility, physicochemical stability, and membrane permeability, processes that directly affect their oral bioavailability [22,23].

CDs are classified into naturals and derivatives. The naturals are subdivided according to the number of glucopyranose units contained in alfa-CD (αCD) (6), beta-CD (βCD) (7), and gamma-CD (γCD) (8), while CD derivatives are formed from natural CDs that underwent substitution reactions in the hydroxyl moieties. Among these are hexakis (2,3,6-tri-O-methyl)-α-CD (TRIMEA), hydroxyethyl-β-CD (HEβCD), hydroxypropyl-β-CD (HPβCD), hydroxypropyl-γ-CD (HPγCD), methylated-β-cyclodextrin (MβCD), randomly methylated-β-cyclodextrin (RAMEB), 2-O-methylated-β-CD (Crysmeb^®^), heptakis (2,6-di-O-methyl)-β-CD (DIMEB), heptakis (2,3,6-tri-O-methyl)-β-CD (TRIMEB), and sulfobutylether-β-CD (SBEβCD) [20,24].

The classical drug:CD complexes are the most studied systems, due to the several types of CDs and manufacturing processes available and the fact that CD complexes could be administered by different routes of administration and incorporated in solid, semisolid and liquid formulations. Notwithstanding, other CD-based systems have been used for drug delivery, including CD nanosponges/polymeric CDs and CD conjugates. Polymeric CDs are formed by the reaction between CDs and a cross-linker substance, providing lower solubility and higher stability than the CDs alone, and allow drug encapsulation by inclusion into CD cavity and noninclusion in the polymer chain [25]. In CD conjugates, the drug is covalently linked to the CD, increasing the stability through its passage in the gastrointestinal tract, being more targeted to drug colon delivery [26].

To perform this review, a literature survey was carried out in different scientific databases, including Scopus, PubMed and ScienceDirect. Initially, all results found until 30 July 2022 were considered, without limiting the search period before this date. The search terms used were a combination of words related to hydroxycinnamic acids (caffeic acid, chlorogenic acid, ferulic acid, coumaric acid, and rosmarinic acid) “AND” cyclodextrin “AND” oral absorption “AND” solubility “AND” stability “AND” release “AND” permeability. Among those results, only research articles in English were considered, and duplicates were disregarded. Additionally, reference lists of papers were screened to detect research papers which did not appear in the database research but might fulfill the acceptance. Afterwards, the papers were screened and selected if meeting the acceptance criteria such as original paper and combining the use of any hydroxycinnamic acid and cyclodextrins.

## 2. Hydroxycinnamic Acids and Cyclodextrin (CD)-Based Delivery Systems

**Figure 2:**
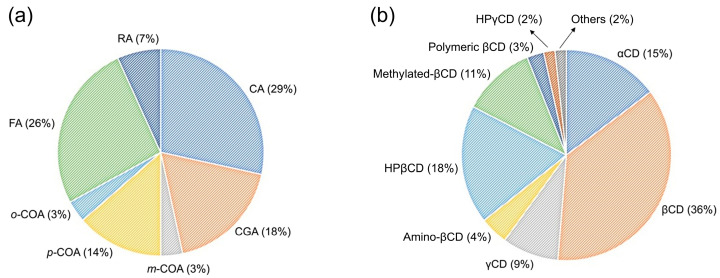
Percentage of studies published with each (**a**) HCA and (**b**) type of CD.

From the data arranged in Table 1 it is possible to observe that several studies comprising HCAs and CDs were found in the literature. CA and FA were the HCAs more investigated, and natural CDs were the type of CDs that presented the greatest number of studies (Figure 2). The higher employment of CD naturals can be associated with the fact that these were the first to be discovered, presenting great complexation capacity and low cost when compared with CD derivatives [27]. The complexation of HCAs with CDs was widely investigated, while conjugation was only applicable for FA with βCD and aminoβCD in two studies [28,29]. Similarly, monomeric CDs were the main type of CDs in the studies, while polymeric βCD was a delivery system only explored three times for CA, CGA, and FA [30,31,32]. Most of the studies investigated the complexation parameters, stoichiometric ratio, and binding constant, using a variety of approaches and experimental conditions. Data are displayed in Table 2 in order to easily assess the information.

## 3. Stoichiometric Ratio and Binding Constant (K) of Hydroxycinnamic Acids and Cyclodextrins 

The determination of stoichiometric ratio and binding constant (*K*) define the degree of interaction between drugs and CDs, being important parameters for the development of oral pharmaceutical dosage forms that contain CD [22,91].

A 1:1 (HCA:CD) stoichiometric ratio is shown for the majority of complexes, with the exception for CA with DIMEB, FA with HPγCD and DIMEB, and RA with HPβCD, which presented 2:1 or 1:2 (CA, FA, or RA:CD) ratios [52,54,78,84,86,90]. The difference in the ratios for RA:HPβCD was attributed to the approach employed for their determination. Veras et al. (2019) [90] determined the ratio by phase-solubility diagram, with excess of RA, above its intrinsic water solubility, while Çelik et al. (2011) [86] and Andreadelis et al. (2020) [52] determined by Benesi–Hildebrand equation and mass analysis, respectively, using an amount of RA below its intrinsic water solubility.

The phase-solubility diagram is the classical approach for the determination of the drug:CD stoichiometric ratio [92]. In methods that use aqueous solutions saturated with the drug, such as phase-solubility diagrams, the formation of higher-order complexes is more likely if compared with those that use diluted solutions, as the molecule is already solubilized [21,90]. Singh et al. (2010) [93] demonstrated clear higher-order complex by phase-solubility diagram in the complexation study of curcumin with HPβCD, while the approaches based on diluted solutions only suggested this type of interaction.

In the studies displayed in Table 2, the Benesi–Hildebrand equation was built from fluorescence, ultraviolet (UV), and nuclear magnetic resonance (NMR) data, in which the response is based on changes in the absorption or emission/excitation intensity and in proton chemical shifts [94]. Therefore, the use of a diluted solution is required to avoid plateau on the detection or excessive shifts with the increase of CD concentration and to minimize the effect of noncomplexed form in the measured analytical signal [42,93,95,96].

Regarding *K*, a great variety of values is noted for HCA with the same type of CD (Table 2). Taking as example the βCD that was the most used CD in the complexation studies, the *K* value reported for the CA, CGA, *m*-COA, *p*-COA, *o*-COA, FA, and RA complexed with βCD ranged between 15–936 [37,40], 14.56–873 [31,58], 232–50,000 [70,71], 160–20,000 [46,71], 380–49,250 [70,72], 87–4090 [74,79], and 79.07–2028 M^−1^ [87,89], respectively. Some factors could explain this range of results found for the HCA:CD complexes, such as degree of substitution of the CD derivatives, stoichiometric ratio of the complexes, approach applied for *K* determination, experimental conditions, and the structural moiety of HCA complexed into CD.

The impact of degree of substitution of the CD derivatives on *K* was suggested for FA:HPβCD (218.5–468 M^−1^), FA:HPγCD (477.5–2490 M^−1^), and RA:HPβCD (267–62,010 M^−1^) complexes (Table 2) [84,90]. A clear parallel cannot be traced due to the lack of data about CD derivatives characteristics in some complexation studies; nevertheless, Schönbeck et al. (2010) [97] reported a decreasing of *K* value for the complexes between bile salts and HPβCD with the increasing degree of substitution of HPβCD. With respect to the stoichiometric ratio, the literature suggests that distinct stoichiometric ratios can culminate in contrasting *K* values [98], evidence that supports the results for FA and RA complexes previous cited [52,78,84,86,89,90].

The approaches for *K* determination can also produce dissimilar results since they are based on different theoretical fundamentals, as described above. Most of the *K* values for HCA:CD complexes were carried out by the phase-solubility diagram or Benesi–Hildebrand equation, while few used Scott’s equation and nonlinear least-square methods (Table 2).

The phase-solubility diagram and Benesi–Hildebrand equation approaches determined for CA:βCD complexes a *K* of 318–587 M^−1^ and 516–936 M^−1^, respectively, indicating similar decimal scale, but a large divergence of values [33,34,35,36,37,39,40]. Aside from this, it was clearly observed that *K* varied within each approach, a fact that was related to the robustness of the analytical methods used to obtain the data need to build the curves [87].

Phase-solubility diagram, Benesi–Hildebrand equation, and Scott’s equation are approaches that assume linearity of the curves. The influence of nonlinear methods on *K* was noticed for CGA:βCD complexes. The complexation of CGA with βCD in aqueous medium with pH 5 pointed out a higher *K* for the Benesi–Hildebrand equation (351.2–663.5 M^−1^) than for the nonlinear least-square method (207.2–277 M^−1^) [61]. The use of nonlinear methods has been suggested as a better choice for *K* determination instead of the Benesi–Hildebrand equation, due to it suffering from a highly biased weighting of points used [87]. An exception for it was observed for the CGA:βCD complexes at pH 7, for which linear and nonlinear methods presented comparable results (*K* = 420 and 465 M^−1^) within standard deviation [64], which propose the effect of the experimental conditions on *K*.

Concerning this topic, among the adopted experimental conditions investigated are temperature, pH, and presence of an organic solvent in the complexation medium. For complexes with βCD analyzed by the phase-solubility method using UV at 25 °C, the respective *K* for CA, *p*-COA, and FA ranged between 318–425, 306–338, and 246–326 M^−1^ [33,34,35], while at 30 °C, *K* were lower than 177 M^−1^ for all HCAs [46]. Likewise, a reduction in *K* was noticed for the complexes of CGA with αCD, βCD, and γCD and RA with βCD and γCD in a larger range of temperatures [31,64,89]. The decrease of *K* is associated with the exothermic character of the complexation phenomenon, in which the rise of temperature decreases the affinity of the HCAs for the CD and, consequently, *K* values [99].

The pH of the aqueous complexation medium is another important factor for determination of *K.* The complexation with CDs involves hydrophobic interactions in which the most hydrophobic or unionized moiety of the drug is inserted into the CD cavity [100,101]. HCAs present acid character and low pKa, being easily ionized with an increase of the pH medium and reducing their affinity for the CD. This statement was visualized in the complexation studies of CGA with αCD, βCD, and γCD [31,64], COA isomers with αCD and βCD [70], and RA with βCD [88] (Table 2), in which the more alkali the medium was, the lower the *K*. Conversely, some studies described an increase of *K* with the enhancing of temperature and pH, but the data are not fully discussed [41,45,55,58,79].

Lastly, the addition of organic solvent in the medium is an approach used to increment the complexation efficiency, due to improving the drug’s intrinsic solubility. The modification in the medium composition produced changes in *K* according to the concentration of the organic solvent [36,37,101]. Kfoury et al. (2019) [36] and Nakhle et al. (2020) [37] evaluated the effect of ethanol and DMSO on the complexation of CA and α, β, and γCD, revealing a decrease in *K* with the addition of the solvents since they increase the hydrophobicity of the medium and weaken the driving force necessary for the inclusion phenomenon. It is confirmed by the absence of cross-peaks in NMR analysis of the complexes containing 45% of ethanol in the complexation medium, which attest no interaction between CA and CDs.

**Figure 3:**
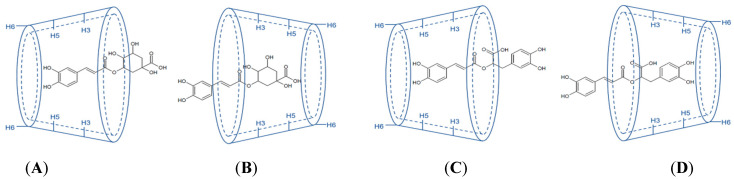
Molecular arrangement of the CGA:βCD (**A**,**B**) and RA:βCD (**C**,**D**) complexes.

With respect to the structural moiety of HCA complexed into CD, CGA and RA, due to their almost symmetric chemical structure, were investigated in the complexation studies with βCD [58,64,87,88]. The investigation of the molecular structure of CGA:βCD complexes at 1:1 ratio by NMR described two probable molecular arrangements. In the first, caffeic acid moiety was included in the βCD cavity, and in the second, the inclusion occurred for the quinic acid moiety (Figure 3A,B) [64]; nonetheless, the structure one appeared as the most thermodynamically stable [48]. This was corroborated by Navarro-Orcajada et al. (2021) [58], who reported a *K* value 21.4-fold lower for the complex between quinic acid moiety of CGA and βCD in comparison with the caffeic acid moiety. For RA, NMR analyses also revealed its two possible arrangements with βCD at a 1:1 ratio, in which both aromatic rings of RA could complex into the βCD cavity (Figure 3C,D) [87,88]. Medronho et al. (2014) [87] found for caffeic acid and 3,4-hydroxyphenyllactic acid moieties of RA complexed with βCD respective *K* of 1184 and 2028 M^−1^, while Aksamija et al. (2016) [88] reported that both complexes are indistinguishable.

The data exposed above demonstrated some examples of studies and factors that could promote changes in the degree of complexation between HCAs with CD, justifying the differences reported. Due to the lack of standardized conditions and the existence of more than one variation on conditions within some studies, a complete correlation among them is made difficult. Moreover, in two complexation studies of CA, *p*-COA, and FA with HPβCD, Crysmeb^®^, and RAMEB, it was observed that the *K* values were quite different, despite the use of the same type of CDs, stoichiometric ratio, method, and experimental conditions [33,34], proposing a possible intravariability in the complexation phenomenon.

## 4. Complexation, Encapsulation, and Loading Efficiencies of Hydroxycinnamic Acid–Cyclodextrin Complexes

As described previously, stoichiometric ratio and *K* values are usually parameters used to measure the degree of interaction and solubilization in complexation systems. However, for some complexes, they do not express the real behavior of the phenomenon since inclusion and noninclusion complexes and water-soluble aggregates could be present at the same time in the solution. In this sense, complexation efficiency (CE) has been assigned as a more precise method to measure the solubilization effectiveness of CDs. Furthermore, it allows to estimate the drug:CD ratio in the complexation medium and the increase in the formulation bulk of a solid dosage form [33,102].

The CE for CA, *p*-COA, and FA with αCD, βCD, HPβCD, RAMEB, and Crysmeb^®^ revealed that αCD presented the highest values for the three HCAs, while βCD showed the lowest results. The molar ratio found for HCA:αCD ranged between 1:1.24 and 1:1.34, which means that one HCA molecule is solubilized by one αCD molecule, promoting the lowest increase in the formulation bulk [33]. For FA, the CE was also obtained with HPγCD, presenting a superior value to the other CDs mentioned above [84].

The presence of ethanol and DMSO (5–45%) in the complexation medium of CA with αCD, βCD, or γCD, in general, promoted a negative effect on CE due to the solvents affecting the medium polarity and complexation driving forces. A positive result was indicated only for the complexes with βCD at 5% of ethanol, which showed higher CE than that with αCD and γCD [36,37]. Based on the data exposed, the use of αCD and the absence of organic solvent are favorable to produce complexes with HCAs aiming at the development of solid dosage forms.

From solid HCA:CD complexes, the encapsulation and loading efficiency data were obtained. The solid complexes between HCA and CD solid complexes were mainly found as freeze-dried (FD) (66.6%), coprecipitated (CP) (25.9%), grounded mixture (GM) (11.1%), spray-dried (SP) (3.7%), and coevaporated (CEva) (3.7%) complexes. A total of 48.1% of the studies prepared physical mixture (PM) of HCA:CD for comparative purposes [28,32,34,35,43,47,51,52,55,56,61,63,65,66,67,68,69,72,73,75,76,80,81,82,83,86,89,90,103].

Encapsulation efficiency (EE) comprises the relationship between the amount of drug in the beginning and the end of the preparation of complexes. RA FD complexes with βCD and γCD exhibited EE > 76% [89]. The FD complexes of CA, *p*-COA, and FA with several types of CDs at 1:1 ratio showed EE values ranging between 61–90%, in which the complexes formed with RAMEB presented the highest results [34]. The influence of *K* on the EE of CA, *p*-COA, and FA and CDs complexes was ruled out since no correlation between *K* and EE was visualized, a fact that was associated with the water solubility of HCAs being above 0.1 mg/mL [33,34].

Aside from *K*, other factors could affect the EE, including concentration of the HCA and CD, the presence of one competitor, CD affinity, and the method for the preparation of complexes [82]. Considering the concentration, FD complexes of CGA with βCD prepared at three different molar ratios, 1:1, 2:1, and 1:3 (CGA:βCD), indicated the respective EE of 26.15, 40.38, and 79.86%. Even though a 1:1 ratio has been established for them, these data demonstrated that the changes in the concentration in one of the components could favor the EE [42,61,63,64,65,66]. In addition, for CGA:βCD solid complexes prepared at 1:1 and 2:1, the molar ratio found was 0.26:1 and 0.81:1, respectively, denoting an increase in CE [65].

The impact of other substances on EE was reported for CA and FA. The CA and FA contained in a vegetal matrix were complexed with βCD, and FA was co-complexed with gallic acid in HPβCD at a 1:1 ratio. The respective EE of CA:βCD, FA:βCD, and FA:HPβCD complexes in the presence of other substances and in isolated form were 19.4 and 63%, 23.2 and 80%, and 68.9 and 68.1% [34,43,82]. Concerning CD affinity, this is a factor influenced by CD physicochemical characteristics. Andreadelis et al. (2020) [52] affirmed that the stability of CA and RA with HPβCD varied according to CD degree of substitution. Similarly, FA SD and FD complexes obtained from HPβCD with different degrees of substitution showed an EE of 60.16 and 80%, respectively [34,81]. The first presented a value close to that found by Olga et al. (2015) [82] for FA:HPβCD FD complexes described above, while the second was significantly higher. Kim (2020) [73] reported for FA:HPβCD FD complexes an EE of 87.74% but the lack of information about HPβCD does not allow a comparison.

Despite the data mentioned above for FA complexed with HPβCD reporting that distinct methods do not lead to divergent EE [81,82], the same was not observed for the FA:αCD complexes. The FA:αCD CP complexes presented an EE of 15.1%, while for the FA:αCD FD complexes, it was 4.83-fold higher. Taking into account that αCD physicochemical characteristics are the same, these results suggest that the method of preparation influences the EE [34,75]. The interaction of FA with polymeric βCD showed an EE ranging, according to the βCD:cross-linker ratio, between 33.33 and 45.75% [32].

Loading efficiency (LE) comprises the relationship between the amount of drug in the complex and the amount of complex. FD complexes of CA, *p*-COA, and FA with αCD, βCD, HPβCD, RAMEB, and Crysmeb^®^ exhibited an LE for all HCA:CD complexes lower than 12.3%. Among these, for each HCA, the CA:RAMEB (10.8%), *p*-COA:αCD (11%), and FA:αCD (12.2%) complexes were the ones that presented the highest LE values [34]. For CGA:βCD FD complexes at 1:1 and 2:1 ratios, the LE for the 2:1 ratio was 2.67-fold (20.12%) higher than for the 1:1 ratio (7.54%), corroborating the EE results [65].

Anselmi et al. (2016) [103] and Wang et al. (2011) [81] reported an LE of 14.99 and 11.05% for FA:γCD CP and FA:HPβCD FD complexes, respectively. A similar LE for FA:HPβCD FD complexes was also found by Kfoury et al. (2016) [34], who reported an LE of 9.2% for the same method of preparation. The LE of FA in the βCD polymer, as described for EE, also ranged, according to the βCD:cross-linker ratio, between 16.85 and 25.7% [32]. For RA, its complexation with βCD, γCD, HPβCD, and Crysmeb^®^ by freeze-drying revealed LE of 24, 23.5, 24.7, and 30%, respectively [89,90]. The studies performed by Budryn et al. (2014) [65] and Rezaei et al. (2019) [32] indicated a correlation between EE and LE, in which the complexes with the highest EE also presented the highest LE. Unlikely, in 9 out of 15 complexes evaluated by Kfoury et al. (2016) [34], EE and LE were not correlated.

## 5. Effect of Cyclodextrins on the Hydroxycinnamic Acids Water Solubility, Dissolution, Release, Stability, and Absorption

**Figure 4:**
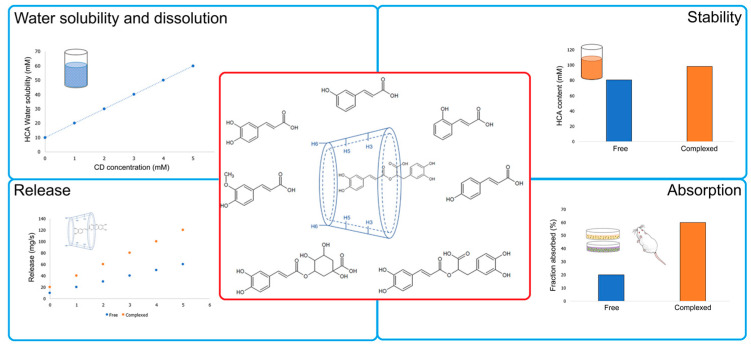
Pathways by which CD complexes could modify the oral bioavailability of drugs.

CD complexes could impact the oral bioavailability of drugs in several forms. In this review, the effect of CD on water solubility, dissolution, release, stability, and absorption is explored (Figure 4).

### 5.1. Water Solubility and Dissolution

The phase-solubility diagram was the first indication of the CD effect on the HCAs water solubility. A linear relationship between CD concentration and HCAs solubilized concentration in water, an A_L_-type profile, was observed for all HCAs [33,35,36,43,44,46,72,78,79,81,84,89,90]. In the presence of αCD, βCD, HPβCD, RAMEB, and Crysmeb^®^ at 10 mM, the improvement in the water solubility for CA, *p*-COA, and FA was 5.5-, 3.0–3.5-, 3.8–4.0-, 4.4-, and 3.8–4.0-fold; 4.8-, 2.8-, 4.4-, 4.4-, and 4.0-fold; and 5.2-, 3.0–3.1-, 4.2–6.0-, 4.8-, and 3.8-fold, respectively [33,34,44,79]. The respective enhancement in FA water solubility at 8 mM of HPβCD and HPγCD was 2.6- and 3.5-fold [84]. For RA, the increase in water solubility by HPβCD and Crysmeb^®^ at 10 mM was 3.33- and 3.47-fold, respectively [90].

Different results of water solubility than these were related for CA, *p*-COA, and FA with αCD, βCD, and HPβCD [43,78,81]. Kalogeropoulos et al. (2009) [43] showed a lower improvement in the water solubility of CA (2.22-fold), *p*-COA (2.25-fold), and FA (2.75-fold) by βCD at 15 mM. For FA, there was improvement in the presence of αCD and HPβCD at 16 mM solubility compared to in these same CDs at 10 mM [33,34,78]. In turn, Wang et al. (2011) [81] reported an increase of 15-fold in the FA water solubility by HPβCD at 8.4 mM.

Most of the studies showed the increase in the water solubility of HCAs by CDs in the phase-solubility diagrams, but only a few demonstrated improvements in the HCA:CD complexes that underwent drying. Han et al. (2019) [28] indicated that the complexation of FA with βCD enhanced its water solubility 2.97-fold, and Rezaei et al. (2019) [32] reported that βCD polymer promoted an enhancement on the water solubility of FA ranging between 3.71–14.48-fold, according to the βCD:cross-linker ratio. These data exhibit a significant difference of CD solubilization capacity on HCAs in dried samples from the phase-solubility data.

The improvement in water solubility of the HCAs was usually related to the formation of their complexes with CDs [28,33,35,36,43,44,46,72,79,81,90], and in the case of βCD polymer, it was also related to the interaction of FA with the pore system structure [32].

The relevance of complexation was evidenced by Han et al. (2019) [28], who compared the effect of βCD conjugation and complexation on the water solubility of FA. As already described, FA:βCD complexes increased the water solubility of FA. On the other hand, FA solubility in the βCD conjugates was lower than the FA alone, a finding associated with the crystalline structure of the conjugated form in water, confirmed by X-ray diffraction analysis.

It is important to mention that the conjugation of FA with some aminoβCD improved its water solubility. The characterization by NMR of the FA conjugated with three types of aminoβCD with different alkyl chains revealed that FA conjugated with the aminoβCD presenting the shorter alkyl chain interacted with the aminoβCD cavity from neighbor conjugates. On the other hand, in FA:aminoβCD conjugates with an intermediary and longer alkyl chain, self-inclusion structures of FA were identified. Despite the inclusion phenomenon, the water solubility of FA for the first conjugate was lower than FA alone, which was associated with the formation of insoluble aggregates due to intermolecular assembly behavior. In contrast, the two last conjugates enhanced the water solubility of FA more than 32-fold, due to self-inclusion phenomena, avoiding the formation of intermolecular packaging. Additionally, X-ray diffraction analysis exhibited amorphous structures for them [29].

Another important aspect ascribed to water solubility is its direct impact on drug dissolution performances. The influence of γCD on the dissolution of CA was evaluated in CA:γCD CP, CA:γCD FD, and CA:γCD GM complexes and CA:γCD PM. CA alone presented a dissolution of 70% at 30 min and reached less than 80% of amount dissolved. In PM form, CA showed a fast dissolution in the first points, but its dissolution profile was similar to CA. On the other hand, CA in CP, FD, and GM complexes showed a fast dissolution in the early stages, reaching almost 100% of dissolution at 30 min [56]. In another study, the dissolution of CA alone was compared with its grounded form and GM complexes and PMs with αCD and βCD. CA alone and its grounded form demonstrated the same slow rate of dissolution, and the respective amounts dissolved at 5 min were 24% and 23%, while the CA:αCD PM, CA:βCD PM, CA:αCD GM, and CA:βCD GM complexes increased the dissolution of CA 1.54-, 2.46-, 4.16-, and 4.16-fold, respectively, for the same period of time [35].

For FA, the dissolution test of FA alone, FA:HPβCD FD complexes, and FA:HPβCD FD PM showed an increase in the dissolution in the following order: FD complexes > PM > FA alone. In the first point, more than 90% of FA in FD complexes form was dissolved, while in alone and FA:HPβCD PM forms, the amount dissolved was similar and lower than 50%. This highest increase of the FA dissolution by FD complexes was correlated to the enhancement of water solubility, verified indirectly by visual analysis of the samples in water. The FA alone and FA:HPβCD PM presented distinct dissolution profiles at 10 min, and the maximum amount dissolved was approximately 50% and 70%, respectively [83].

These findings demonstrated that the simple mixing of CA and FA with CDs in PM form was capable of changing the crystallinity and wettability of CA and FA, at least with αCD, βCD, and HPβCD. However, the methods which use a solvent, such as coprecipitation and freeze-drying, or mechanical energy, such as grinding mixture, promoted a higher increase in their dissolution, due to the formation of complexes [35,56,83]. The choice of complexes, rather than PM, has already been reported as the best technological approach to increase the oral bioavailability of drugs [22].

As complexation with α, β, and γCD showed similar results on the CA dissolution, the selection of the type of CD and process of complexation will depend on the cost associated with each raw material and manufacturing process.

### 5.2. Release

Complexes with CDs can modify the release behavior of drugs from formulation, promoting delayed, prolonged, or sustained profile [104]. CA:HPβCD and FA:αCD complexes, and CA and FA in polymeric βCD, showed the impact of CDs on CA and FA releases [32,51,57,75]. The release of CA and CA:HPβCD solutions were carried out by the dialysis membrane, and for FA and FA:αCD complexes into oil/water emulsion, the release was carried out by the Strainer cell model. A slower release was observed for CA and FA in their complexed forms when compared with their free forms [51,75]. CA solution presented a fast release after 4 h (97.8%), while its complexed form released 93.6% after 24 h [75]. For FA, the amount of its free form released was 2.58-fold higher than the complexed form after 7 h [75]. βCD polymer promoted a slow release of CA and FA; however, the isolated forms of the HCAs were not tested as control [32,57].

### 5.3. Stability

With respect to the impact of complexation on gastrointestinal stability, only one study was found in the literature. The enzymatic digestion of CGAs alone and CGAs:βCD complexes present in foods promoted a recovery of CGAs in free and complexed forms between 86.48–99.12% and 92.46–100%, respectively, indicating that the complexed forms had lower interaction with digestive enzymes and a that higher amount was available for absorption [67]. Regardless of the lack of more data about gastrointestinal stability, some studies investigated the stability of HCA:CD complexes against storage stability, photolysis, and temperature.

The storage stability of CGA complexed with βCD in solution at room temperature was increased in 4 weeks when compared CGA alone [63]. Light and temperature have no effect on the processes of oral absorption of drugs, but they are important to ensure the quality of the formulation during manufacture, storage, and use [105,106]. *p*-COA and FA underwent cis-isomerization in UV-A irradiation or sunlight exposition in a short period of time, while CA, CGA, and RA are quite stable, presenting minimum formation of cis-isomers [107,108].

FA degradation under UV-B irradiation showed the first-order kinetics with a rate constant of 0.0579 h^−1^. Its complexation with HPβCD reduced the amount degraded and rate constant 1.71- and 5-fold, respectively [81]. FA:αCD complexes into oil/water emulsion fully prevented FA degradation by UV-B irradiation, while the remaining content of FA alone in emulsified form was 69.60%. The solution of FA was not tested as control; therefore, it is not possible to affirm whether protection was due exclusively to complexation or an additive effect with emulsion [75].

The photostability of FA alone and of its complexed and conjugated forms with βCD under UV-B irradiation followed the order: conjugates (77%) > complexes (42%) > FA alone (33%) [28]. FA:aminoβCD conjugates revealed that the protection was dependent on the alkyl chain size of amino-βCD, in which the intermediary chain (72%) presented higher stability than the shorter (58%) and longer (53%) chains [29]. The highest stabilization promoted by the conjugates was associated with the esterification of FA on βCD and aminoβCDs that decreased its isomerization [28]. The photostability of the RA against UV-C irradiation was increased by its complexation with βCD and γCD, exhibiting an apparent pseudo-first-order rate constant 2.04- and 1.61-fold lower than RA alone [89].

There are no specific data about the thermal stability of FA, but the complexes had a substantial impact on it. Differential thermal analysis and thermogravimetric analyses of FA showed that its first event of decomposition starts at 170–177 °C with a mass loss of 90% [28,109]. FA:βCD complexes and conjugates increased the temperature of the decomposition event and decreased the mass loss to 81% and 72%, respectively. A more pronounced loss of mass was observed for the FA conjugates with aminoβCDs, which was reduced to values ranging from 32–65%. The higher enhancement in the thermal stability of FA by conjugation than the complexation was due to the formation of strong intermolecular linkages in the conjugates when compared with the noncovalent bonding in the complexes [28,29].

### 5.4. In Vitro and In Vivo Absorption Studies

In addition to the factors described above, the oral absorption of drugs is also dependent on their permeability through the unstirred water layer and gastrointestinal membranes. Several in vitro and in vivo studies reported CDs as permeability enhancers due to them affecting both structures, allowing the passage of the drugs through the barriers [23].

A permeability study in Caco-2 cells of CA and *p*-COA present in alpujero, a two-phase olive mill waste, complexed with βCD revealed a slight increase for *p*-COA in the intracellular (0.2%) and transported amounts toward the basolateral side (8.5%) when compared to uncomplexed *p*-COA. CA was not detected intracellularly or in the basolateral side. This disappearance of CA was associated with its metabolism by COMT since FA was found in the Caco-2 intracellular space and basolateral side. Furthermore, it was observed that the transport rate of FA toward the basolateral side of Caco-2 cells increased 1.25-fold in the presence of βCD [47].

A nonquantitative analysis, based on the intensity of fluorescence, indicated an improvement of the FA cellular uptake by Hep3B cells when treated with FA:HPβCD complexes. The authors reported this improvement to the higher water solubility of FA in complexed form. Nonetheless, the FA concentration tested (200 µM; ~0.04 mg/mL) is under its intrinsic solubility [110], and manipulation effect of HPβCD on the Hep3B cells permeability cannot be ruled out. Additionally, in vivo oxidative stress induced by CCl_4_ study demonstrated that the oral treatment with FA:HPβCD complexes decreased the levels of alanine aminotransferase, aspartate aminotransferase, and malondialdehyde and significantly increased the levels of superoxide dismutase in comparison with FA alone. Regardless of the lack of pharmacokinetic data, these results together suggested that complexation promoted higher absorption of FA, which increased the protection for the liver [83].

Another study, with rats that underwent oxidative stress through a high-fat diet fed with CGAs complexed with βCD, showed that the enhancement of the antioxidant capacities of plasma water and lipid fractions and the decreased level of thiobarbituric acid reactive substances were significantly better than those fed only with CGAs. The same results were observed for the groups that did not undergo oxidative stress. These findings indicated that the complexation increased bioaccessibility and absorbed amount of CGAs [68].

The results presented reveal that CD complexation represents a great technological approach to improve the parameters that impact the oral bioavailability of HCAs. In order to easily access the main results, a summary is described in Table 3.

## 6. Conclusions

In studies of CD-based delivery systems containing HCAs, CA and FA were the most explored, and the majority focused on the analysis of the interactions between HCAs and CDs, revealing that several factors can affect the degree of interaction of the systems, as well as the complexation, encapsulation, and loading efficiencies, factors that could be criteria of selection for researchers and industry to produce the best cost–benefit CD-based delivery system. Complexation systems were extensively investigated in comparison to conjugation, and few studies employed polymeric CDs.

Regarding the effects of CD-based delivery systems on fundamental parameters for oral bioavailability, positive effects are reported on solubility, both in phase-solubility studies and in solid complexes. Despite few studies investigating the influence of CD on stability and absorption of HCAs, the results found denote that CD complexation is a favorable technological approach to improve the performance of HCAs in these parameters.

The presented data emphasize a need for more studies concerning the processes involved in the oral administration of HCAs from CD-based delivery, which remains an underexplored field despite the promising results.

## Figures and Tables

**Table 1:** Studies in the literature reporting the interaction of HCAs with CDs.

HCA	Type of CD	References
CA	αCD	[30,33,34,35,36,37,38]
	βCD	[30,33,34,35,36,37,38,39,40,41,42,43,44,45,46,47,48,49]
	HPβCD	[30,33,34,44,45,50,51,52]
	SBEβCD	[53]
	MβCD	[30,45]
	Crysmeb^®^	[33,34]
	DIMEB	[54]
	RAMEB	[33,34]
	γCD	[30,36,55,56]
	Polymeric βCD	[57]
CGA	αCD	[31,58]
	βCD	[31,40,42,48,58,59,60,61,62,63,64,65,66,67,68]
	HPβCD	[58,60,66,69]
	MβCD	[58]
	γCD	[31,58]
	Polymeric βCD	[31]
*m*-COA	αCD	[70,71]
	βCD	[70,71,72]
*o*-COA	αCD	[70,71]
	βCD	[70,71,72]
*p*-COA	αCD	[33,34,70,71]
	TRIMEA	[54]
	βCD	[33,34,43,46,47,49,70,71,72]
	HPβCD	[33,34,50,73]
	Crysmeb^®^	[33,34]
	DIMEB	[54]
	RAMEB	[33,34]
	TRIMEB	[54]
FA	αCD	[33,34,38,74,75,76,77,78]
	TRIMEA	[54]
	βCD	[28,33,34,38,43,46,49,74,77,79,80]
	AminoβCD	[29,74]
	HPβCD	[33,34,73,78,79,81,82,83,84]
	SBEβCD	[53]
	MβCD	[78]
	Crysmeb^®^	[33,34]
	DIMEB	[54]
	RAMEB	[33,34]
	γCD	[74,77,85]
	HPγCD	[78,84]
	Polymeric βCD	[32]
RA	αCD	[86]
	βCD	[86,87,88,89]
	HEβCD	[86]
	MβCD	[86]
	Crysmeb^®^	[90]
	HPβCD	[52,86,90]
	γCD	[89]

**Table 2:** Parameters of the complexation studies of HCAs with CDs for determination of the stoichiometric ratio and binding constant and their results.

HCA	CD	Approaches Used for Ratio and/or *K* Determination	Analytical Methods	Ratio (HCA:CD)	Binding Constant (*K*) (M^−1^)							References
CA	βCD	Benesi–Hildebrand equation	UV	1:1	516							[39]
CA CGA	βCD	Job’s plot Benesi–Hildebrand equation	NMR	1:1	CA: 936 CGA: 504							[40]
CA	βCD	Benesi–Hildebrand equation	Fluorescence	1:1	268 (pH 3.05) 253 (pH 7.5) 475 (pH 10.53) 73 (pH 12.5)							[41]
CA CGA	βCD	Benesi–Hildebrand equation	Fluorescence	1:1	CA: 278 (pH 7) CGA: 424 (pH 7)							[42]
CA	βCD HPβCD	Benesi–Hildebrand equation Phase-solubility diagram	Fluorescence UV	1:1	βCD: Not expressed			HPβCD 112 (Water) 580 (pH 3) 279 (pH 6.5) 104 (pH 10.5)				[44]
CA	γCD	Benesi–Hildebrand equation	Fluorescence UV	1:1	943 57.5 (pH 3.05) 168.5 (pH 5) 377.1 (pH 6.5) 1430 (pH 8.96)			52.4 (25 °C) 113.8 (30 °C) 208.5 (37 °C) 84.3 (45 °C)				[55]
CA *p*-COA FA	αCD βCD HPβCD (DS 5.6) RAMEB (DS 12.6) MβCD (Crysmeb^®^) (DS 4.9)	Phase-solubility diagram	UV	1:1	CA	*p*-COA		FA				[33]
					αCD: 1819	αCD: 1988		αCD: 1737				
					βCD: 425	βCD: 306		βCD: 326				
					HPβCD: 534	HPβCD: 1099		HPβCD: 833				
					RAMEB: 825	RAMEB: 1228		RAMEB: 1045				
					Crysmeb^®^: 552	Crysmeb^®^: 900		Crysmeb^®^: 512				
CA FA	SBEβCD (DS 7.0)	Double reciprocal plot	Chemiluminescence	1:1	CA: 18,600.00 FA: 47,900.00							[53]
CA	βCD HPβCD (MS 0.6) MβCD (MS 1.6)	Benesi–Hildebrand equation	UV	1:1	βCD: 133 (pH 3) 178 (pH 5)	HPβCD: 10 (pH 3) 37 (pH 5)		MβCD: Not expressed				[45]
CA *p*-COA FA	αCD βCD HPβCD (DS 5.6) RAMEB (DS 12.6) MβCD (Crysmeb^®^) (DS 4.9)	Phase-solubility diagram	UV	1:1	CA αCD: 1540 βCD: 318 HPβCD: 526 RAMEB: 991 Crysmeb^®^: 404	*p*-COA αCD: 1816 βCD: 338 HPβCD: 787 RAMEB: 1030 Crysmeb^®^: 668		FA αCD: 1769 βCD: 246 HPβCD: 451 RAMEB: 908 Crysmeb^®^: 474				[34]
CA *p*-COA FA	βCD	Phase-solubility diagram	UV	1:1	CA: 176 *p*-COA: 160 FA: 133							[46]
CA FA	αCD βCD	Benesi–Hildebrand equation	Fluorescence UV	1:1	Fluorescence			UV				[38]
					CA αCD: 387 (pH 7) βCD: 431 (pH 7)	FA αCD: 479 (pH 7) βCD: 625 (pH 7)		CA αCD: 288 (pH 7) βCD: 363 (pH 7)		FA αCD: 249 (pH 7) βCD: 541 (pH 7)		
CA	αCD βCD	Phase-solubility diagram	HPLC	1:1	αCD: 1547.5 βCD: 371.4							[35]
CA	αCD βCD γCD	Phase-solubility diagram	UV	1:1	αCD	βCD		γCD				[36]
					1512 (water)	390 (water)		297 (water)				
					256 (5% ethanol)	363 (5% ethanol)		190 (5% ethanol)				
					74 (15% ethanol)	174 (15% ethanol)		89 (15% ethanol)				
					27 (25% ethanol)	44 (25% ethanol)		42 (25% ethanol)				
					7 (35% ethanol)	19 (35% ethanol)		11 (35% ethanol)				
CA RA	HPβCD	Mass analysis Titration	ESI–MS ITC	1:1	CA: 760 RA: 1800							[52]
CA	αCD βCD	Phase-solubility diagram	UV	1:1	αCD			βCD				[37]
					1463 (water)			587 (water)				
					770 (1% ethanol)			383 (1% ethanol)				
					295 (5% ethanol)			327 (5% ethanol)				
					136 (15% ethanol)			234 (15% ethanol)				
					20 (25% ethanol)			163 (25% ethanol)				
					1176 (1% DMSO)			540 (1% DMSO)				
					736 (5% DMSO)			293 (5% DMSO)				
					235 (15% DMSO)			153 (15% DMSO)				
					109 (25% DMSO)			65 (25% DMSO)				
					56 (35% DMSO)			36 (35% DMSO)				
					21 (45% DMSO)			15 (45% DMSO)				
CGA	αCD βCD γCD Polymeric βCD	Job’s plot	NMR	1:1	αCD 509 (pH 3.6/3 °C) 426 (pH 3.6/13 °C) 321 (pH 3.6/25 °C) 249 (pH 3.6/37 °C) 1144 (pH 6.5/3 °C) 887 (pH 6.5/13 °C) 626 (pH 6.5/25 °C) 446 (pH 6.5/37 °C)	βCD 873 (pH 3.6/3 °C) 672 (pH 3.6/13 °C) 526 (pH 3.6/25 °C) 416 (pH 3.6/37 °C) 799 (pH 6.5/3 °C) 663 (pH 6.5/13 °C) 597 (pH 6.5/25 °C) 468 (pH 6.5/37 °C)		γCD 555 (pH 3.6/3 °C) 412 (pH 3.6/13 °C) 400 (pH 3.6/25 °C) 46 (pH 6.5/3 °C) 31 (pH 6.5/13 °C) 16 (pH 6.5/25 °C)			Polymeric βCD 332 (pH 3.6/3 °C) 428 (pH 3.6/13 °C) 501 (pH 3.6/25 °C) 499 (pH 3.6/37 °C) 509 (pH 3.6/40 °C) 360 (pH 3.6/50 °C) 297 (pH 3.6/60 °C) 197 (pH 6.5/3 °C) 422 (pH 6.5/13 °C) 544 (pH 6.5/25 °C) 570 (pH 6.5/37 °C) 593 (pH 6.5/40 °C) 552 (pH 6.5/50 °C) 330 (pH 6.5/60 °C)	[31]
CGA	βCD	Nonlinear least-squares method Benesi–Hildebrand equation	Fluorescence	1:1	Nonlinear 465 (pH 7)			Benesi–Hildebrand equation 420 (pH 7)				[61]
CGA	βCD	Nonlinear least-squares method Benesi-Hildebrand equation	Fluorescence	1:1	Nonlinear 277 (5 °C/pH 5) 260.3 (10 °C/pH 5) 253.2 (15 °C/pH 5) 207.2 (25 °C/pH 5)			Benesi–Hildebrand equation 663.5 (5 °C/pH 5) 504.3 (10 °C/pH 5) 390.8 (5 °C/pH 5) 351.2 (25 °C/pH 5)				[64]
CGA	HPβCD	Benesi-Hildebrand equation	Fluorescence	1:1	155.7 (pH 5)							[69]
CGA	αCD βCD γCD HPβCD (DS 5) MβCD (DS 5.4)	-	Fluorescence	1:1	αCD 20.83–203.66 (25 °C/pH 3) 32.63–530.06 (25 °C/pH 5) 35.68–757.86 (25 °C/pH 9)	βCD 15.37–286.59 (25 °C/pH 3) 14.56–311.75 (25 °C/pH 5) 16.38–170.71 (25 °C/pH 9)	γCD 6.46–20.53 (25 °C/pH 3) 0.58 (25 °C/pH 5) 5.55–25.26 (25 °C/pH 9)	HPβCD 23.56–471.22 (25°C/pH 3) 21.20–439.52 (25 °C/pH 5) 16.47–163.31 (25 °C/pH 9)			MβCD 19.42–397.49 (25 °C/pH 3) 20.27–381.87 (25 °C/pH 5) 19.41–389.86 (25 °C/pH 9)	[58]
*m*-COA *o*-COA *p*-COA	αCD βCD	Job’s plot Scott’s equation	UV	1:1	*m*-COA	*o*-COA		*p*-COA				[70]
					αCD: 1320 (pH 1.6)	αCD: 1100 (pH 1.6)		αCD: 1990 (pH 1.6)				
					αCD: 90 (pH 8.2)	αCD: Not expressed (pH 8.2)		αCD: 110 (pH 8.2)				
					βCD: 426 (pH 1.6)	βCD: 380 (pH 1.6)		βCD: 570 (pH 1.6)				
					βCD: 232 (pH 8.2)	βCD: Not expressed (pH 8.2)		βCD: 412 (pH 8.2)				
*m*-COA *o*-COA *p*-COA	βCD	Phase-solubility diagram	HPLC	1:1	*m*-COA: 390 *o*-COA: 49,250 *p*-COA: 2810							[72]
*m*-COA *o*-COA *p*-COA	αCD βCD	Mass analysis	ESI–MS	1:1	*m*-COA	*o*-COA			*p*-COA			[71]
					αCD:	α*CD:*			α*CD:*			
					20,000–40,000 (pH 4–5)	3000–11,000 (pH 4–5)			20,000–50,000 (pH 4–5)			
					βCD:	β*CD:*			β*CD:*			
					11,000–50,000 (pH 4–5)	6000–22,000 (pH 4–5)			6000–20,000 (pH 4–5)			
FA	αCD	Nonlinear least-squares method	Fluorescence	1:1	αCD: 1113 (pH 7.2)							[74]
	βCD				βCD: 4090 (pH 7.2)							
	γCD				γCD: 707 (pH 7.2)							
	NH_2_(CH_2_)_2_NHβCD				NH_2_(CH_2_)_2_NHβCD: 1580 (pH 7.2)							
	NH_2_(CH_2_)_2_NH(CH_2_)_2_NHβCD				NH_2_(CH_2_)_2_NH(CH_2_)_2_NHβCD: 356 (pH 7.2)							
FA	αCD	Job’s plot Nonlinear regression method	NMR	1:1	1162 (pH 4)							[75]
FA	βCD HPβCD	Benesi–Hildebrand equation Phase-solubility diagram	Fluorescence	1:1	βCD			HPβCD				[79]
					87			98				
					102 (pH 3.05)			128 (pH 3.05)				
					205 (pH 7.5)			590 (pH 7.5)				
					Not expressed (pH 10.53)			93 (pH 10.53)				
FA	HPβCD	Phase-solubility diagram	HPLC	1:1	166.3							[81]
FA	αCD	Nonlinear regression method	ITC	-	αCD: 53.2 (pH 9)							[77]
	βCD				βCD: 176.5 (pH 9)							
	γCD				γCD: 19.4 (pH 9)							
FA	αCD	Phase-solubility diagram Job’s plot	HPLC UV	1:1 (αCD, MβCD, and HPβCD) 2:1 (HPγCD)	αCD: 250							[78]
	MβCD				MβCD: 238							
	HPβCD				HPβCD: 218.5							
	HPγCD				HPγCD: 477.5							
FA	HPβCD (DS ~0.9)	Phase-solubility diagram	UV	1:1	HPβCD: 468							[84]
	HPγCD (DS ~0.6)				HPγCD: 2490							
RA	αCD	Benesi–Hildebrand equation	Fluorescence	1:1	αCD: 82 (pH7)							[86]
	βCD				βCD: 164 (pH7)							
	HEβCD				HEβCD: 168 (pH7)							
	HPβCD				HPβCD: 267 (pH7)							
	MβCD				MβCD: 328 (pH7)							
RA	βCD	Job’s plot Nonlinear least-square method	NMR	1:1	βCD:1184–2028 (pH 7.8)							[87]
RA	βCD	Job’s plot Scott’s equation Nonlinear method	NMR CE	1:1	Scott’s plot 300–468 (pH 1) 260–393 (pH 2.9) 202–319 (pH 6)			Nonlinear 176 and 197 (pH 7)				[88]
RA	HPβCD (DS 0.8) MβCD (Crysmeb^®^) (DS 0.57)	Phase-solubility diagram	HPLC	2:1	HPβCD: 62,010 Crysmeb^®^: 61,454							[90]
RA	βCD γCD	Phase-solubility diagram	UV	1:1	βCD			γCD				[89]
					109.79 (15 °C/pH 7.4)			88.70 (15 °C/pH 7.4)				
					100.46 (18 °C/pH 7.4)			81.55 (18 °C/pH 7.4)				
					86.70 (21 °C/pH 7.4)			70.26 (21 °C/pH 7.4)				
					79.07 (25 °C/pH 7.4)			63.62 (25 °C/pH 7.4)				

CA: caffeic acid. CGA: chlorogenic acid. COA: coumaric acid. FA: ferulic. RA: rosmarinic acid. CE: capillary electrophoresis. DS: degree of substitution. ESI–MS: electrospray ionization–mass spectroscopy. HPLC: high-performance liquid chromatography. ITC: isothermal titration calorimetry. MS: molar substitution. NMR: nuclear magnetic resonance. UV: ultraviolet spectroscopy. αCD alfa-CD. βCD: beta-CD, γCD: gamma-CD. HEβCD: hydroxyethyl-β-CD. HPβCD: hydroxypropyl-β-CD. HPγCD: hydroxypropyl-γ-CD. MβCD: methylated-β-cyclodextrin. RAMEB: randomly methylated-β-cyclodextrin. Crysmeb^®^: 2-O-methylated-β-CD. SBEβCD: sulfobutylether-β-CD. NH2(CH2)2NHβCD: Mono [6-(2-aminoethyleneamino)-6-deoxy]-β-cyclodextrin. NH2(CH2)2NH(CH2)2NHβCD: mono [6-(5-amino-3-azapentylamino)- 6-deoxy]-β-cyclodextrin. Both 1:1 and 2:1 ratios of RA:HPβCD complexes in solution were confirmed by electrospray ionization coupled with mass spectroscopy (ESI–MS) [52,90], but it is worth mentioning that the RA:HPβCD complexes studied by Andreadelis et al. (2020) [52] were prepared at an initial ratio of 1:2 (RA:HPβCD) and this ratio was not justified. For FA:HPγCD complexes, even though 1:1 and 2:1 stoichiometric ratios have been achieved by the phase-solubility technique, apparently, the 2:1 ratio was not based on the slope of the curve, but on the capacity of solubilization of HPγCD in the highest concentration, which was twice as high as the other CDs, while in the complexation of FA and CA with DIMEB the 1:2 ratio was based on the loss of water in the thermogravimetric analysis [54,78,84].

**Table 3:** Summary of the main results concerning the parameters that affect the oral bioavailability of HCAs presented in the review.

HCA	CD	Results			References
CA	βCD HPβCD	Improvement of water solubility in phase-solubility assay *.			[44]
CA *p*-COA FA	αCD βCD HPβCD (DS 5.6) RAMEB (DS 12.6) MβCD (Crysmeb^®^) (DS 4.9)	Improvement of water solubility (CD at 10 mM) (phase-solubility assay):			[33]
		CA	*p*-COA	FA	
		αCD: 5.5-fold	αCD: 4.8-fold	αCD: 5.2-fold	
		βCD: 3.5-fold	βCD: 2.8-fold	βCD: 3.1-fold	
		HPβCD: 3.8-fold	HPβCD: 4.4-fold	HPβCD: 4.4-fold	
		RAMEB: 4.4-fold	RAMEB: 4.4-fold	RAMEB: 4.8-fold	
		MβCD: 3.8-fold	MβCD: 4.0-fold	MβCD: 3.8-fold	
CA *p*-COA FA	αCD βCD HPβCD (DS 5.6) RAMEB (DS 12.6) MβCD (Crysmeb^®^) (DS 4.9)	Improvement of water solubility (CD at 10 mM) (phase-solubility assay):			[34]
		CA	*p*-COA	FA	
		αCD: 5.5-fold	αCD: 4.8-fold	αCD: 5.2-fold	
		βCD: 3.5-fold	βCD: 2.8-fold	βCD: 3.1-fold	
		HPβCD: 3.8-fold	HPβCD: 4.4-fold	HPβCD: 4.4-fold	
		RAMEB: 4.4-fold	RAMEB: 4.4-fold	RAMEB: 4.8-fold	
		MβCD: 3.8-fold	MβCD: 4.0-fold	MβCD: 3.8-fold	
CA *p*-COA FA	βCD	Improvement of water solubility in phase-solubility assay *.			[46]
CA	αCD	Improvement of water solubility in phase-solubility assay *.			[35]
		Enhance of dissolution:			
		CA:αCD PM: 1.54-fold			
	βCD	CA:αCD GM: 4.16-fold			
		CA:βCD PM: 2.46-fold			
		CA:βCD GM: 4.16-fold			
CA	αCD	Improvement of water solubility in phase-solubility assay *.			[36]
	βCD				
	γCD				
CA	αCD	Improvement of water solubility in phase-solubility assay *.			[37]
	βCD				
*m*-COA *o*-COA *p*-COA	βCD	Improvement of water solubility in phase-solubility assay *.			[72]
FA	βCD	Improvement of water solubility in phase-solubility assay *.			[79]
	HPβCD				
FA	HPβCD	Improvement of water solubility (CD at 8.4 mM) (phase-solubility assay): HPβCD: 15-fold			[81]
FA	αCD MβCD HPβCD HPγCD	Improvement of water solubility (CD at 16 mM) (phase-solubility assay):			[78]
		αCD: 5.0-fold			
		MβCD: 4.8-fold			
		HPβCD: 4.5-fold			
		HPγCD: 8.3-fold			
FA	HPβCD (DS ~0.9) HPγCD (DS ~0.6)	Improvement of water solubility (CD at 8 mM) (phase-solubility assay):			[84]
		HPβCD: 2.6-fold			
		HPγCD: 3.5-fold			
RA	HPβCD (DS 0.8) MβCD (Crysmeb^®^) (DS 0.57)	Improvement of water solubility (CD at 10 mM) (phase-solubility assay):			[90]
		HPβCD: 3.33-fold			
		MβCD: 3.47-fold			
RA	βCD γCD	Improvement of water solubility in phase-solubility assay *.			[89]
FA	βCD	Improvement of water solubility in solid complex: 2.29-fold			[28]
FA	βCD polymer	Improvement of water solubility in solid complex: 3.71–14.48-fold			[32]
CA	γCD	Increase of dissolution: CA:γCD PM: Profile similar to CA (less than 80% dissolved at 120 min) CA:γCD CP, FD, and GM: 100% dissolved at 30 min			[56]
FA	HPβCD	Increase of dissolution:			[83]
		FA:HPβCD PM: 70% dissolved at 30 min			
		FA:HPβCD FD: 90% dissolved at ~10 min			
CGA	βCD	Maintenance of stability: 92.46–100% of CGA content remaining.			[67]
*p*-COA	βCD	Improvement of intracellular accumulation of *p*-COA and its transport toward basolateral side in Caco-2 cells.			[47]
FA	HPβCD	Increase of intracellular accumulation of FA in Hep3B cells.			[83]
CGA	βCD	Enhancement of the pharmacological activity which was attributed to the improvement of absorption.			[68]

CA: caffeic acid. CGA: chlorogenic acid. *p*-COA: para-coumaric acid. FA: ferulic. αCD alfa-CD. βCD: beta-CD, γCD: gamma-CD. HPβCD: hydroxypropyl-β-CD. HPγCD: hydroxypropyl-γ-CD. MβCD: methylated-β-cyclodextrin. RAMEB: randomly methylated-β-cyclodextrin. FD: freeze-dried. CP: coprecipitated. GM: grounded mixture. PM: physical mixture. * Data shown only graphically.

## Data Availability

All data are provided in the manuscript or cited in the references.

## References

[1] Kumar N., Goel N. (2019). Phenolic Acids: Natural Versatile Molecules with Promising Therapeutic Applications. Biotechnol. Rep..

[2] Lafay S., Gil-Izquierdo A. (2008). Bioavailability of Phenolic Acids. Phytochem. Rev..

[3] Rashmi H.B., Negi P.S. (2020). Phenolic Acids from Vegetables: A Review on Processing Stability and Health Benefits. Food Res. Int..

[4] Coman V., Vodnar D.C. (2020). Hydroxycinnamic Acids and Human Health: Recent Advances. J. Sci. Food Agric..

[5] Sova M., Saso L. (2020). Natural Sources, Pharmacokinetics, Biological Activities and Health Benefits of Hydroxycinnamic Acids and Their Metabolites. Nutrients.

[6] Zhao Z., Moghadasian M.H. (2010). Bioavailability of Hydroxycinnamates: A Brief Review of in vivo and in vitro Studies. Phytochem. Rev..

[7] Granata G., Consoli G.M.L., Lo Nigro R., Geraci C. (2018). Hydroxycinnamic Acids Loaded in Lipid-Core Nanocapsules. Food Chem..

[8] Chen L., Liu C.S., Chen Q.Z., Wang S., Xiong Y.A., Jing J., Lv J.J. (2017). Characterization, Pharmacokinetics and Tissue Distribution of Chlorogenic Acid-Loaded Self-Microemulsifying Drug Delivery System. Eur. J. Pharm. Sci..

[9] Liu C.S., Chen L., Hu Y.N., Dai J.L., Ma B., Tang Q.F., Tan X.M. (2020). Self-Microemulsifying Drug Delivery System for Improved Oral Delivery and Hypnotic Efficacy of Ferulic Acid. Int. J. Nanomed..

[10] Zhang Y., Li Z., Zhang K., Yang G., Wang Z., Zhao J., Hu R., Feng N. (2016). Ethyl Oleate-Containing Nanostructured Lipid Carriers Improve Oral Bioavailability of Trans-Ferulic Acid Ascompared with Conventional Solid Lipid Nanoparticles. Int. J. Pharm..

[11] Madureira A.R., Campos D.A., Oliveira A., Sarmento B., Manuela M., Maria A. (2016). Insights into the Protective Role of Solid Lipid Nanoparticles on Rosmarinic Acid Bioactivity during Exposure to Simulated Gastrointestinal Conditions. Colloids Surf. B Biointerfaces.

[12] Nallamuthu I., Devi A., Khanum F. (2015). Chlorogenic Acid Loaded Chitosan Nanoparticles with Sustained Release Property, Retained Antioxidant Activity and Enhanced Bioavailability. Asian J. Pharm. Sci..

[13] Yang J.H., Zhang L., Li J.S., Chen L.H., Zheng Q., Chen T., Chen Z.P., Fu T.M., Di L.Q. (2015). Enhanced Oral Bioavailability and Prophylactic Effects on Oxidative Stress and Hepatic Damage of an Oil Solution Containing a Rosmarinic Acid-Phospholipid Complex. J. Funct. Foods.

[14] Yang J.-H., Mao K.-J., Huang P., Ye Y.-J., Guo H.-S., Cai B.-C. (2018). Effect of Piperine on the Bioavailability and Pharmacokinetics of Rosmarinic Acid in Rat Plasma Using UPLC-MS/MS. Xenobiotica.

[15] Jung J.W., Kim J.M., Jeong J.S., Son M., Lee H.S., Lee M.G., Kang H.E. (2014). Pharmacokinetics of Chlorogenic Acid and Corydaline in DA-9701, a New Botanical Gastroprokinetic Agent, in Rats. Xenobiotica.

[16] Pinho E., Grootveld M., Soares G., Henriques M. (2014). Cyclodextrins as Encapsulation Agents for Plant Bioactive Compounds. Carbohydr. Polym..

[17] Suvarna V., Gujar P., Murahari M. (2017). Complexation of Phytochemicals with Cyclodextrin Derivatives—An Insight. Biomed. Pharmacother..

[18] Adeoye O., Cabral-Marques H. (2017). Cyclodextrin Nanosystems in Oral Drug Delivery: A Mini Review. Int. J. Pharm..

[19] Brewster M.E., Loftsson T. (2007). Cyclodextrins as Pharmaceutical Solubilizers. Adv. Drug Deliv. Rev..

[20] Loftsson T., Brewster M.E. (2010). Pharmaceutical Applications of Cyclodextrins: Basic Science and Product Development. J. Pharm. Pharmacol..

[21] Loftsson T., Másson M., Brewster M.E. (2004). Self-Association of Cyclodextrins and Cyclodextrin Complexes. J. Pharm. Sci..

[22] Carrier R.L., Miller L.A., Ahmed I. (2007). The Utility of Cyclodextrins for Enhancing Oral Bioavailability. J. Control. Release.

[23] Loftsson T., Vogensen S.B., Brewster M.E., Konrasdottir F. (2007). Effects of Cyclodextrins on Drug Delivery through Biological Membranes. J. Pharm. Sci..

[24] Szente L., Szejtli J. (1999). Highly Soluble Cyclodextrin Derivatives: Chemistry, Properties, and Trends in Development. Adv. Drug Deliv. Rev..

[25] Sherje A.P., Dravyakar B.R., Kadam D., Jadhav M. (2017). Cyclodextrin-Based Nanosponges: A Critical Review. Carbohydr. Polym..

[26] Shahiwala A. (2020). Cyclodextrin Conjugates for Colon Drug Delivery. J. Drug Deliv. Sci. Technol..

[27] Da Cunha Filho M.S.S., Sá-Barreto L.C.L. (2007). Utilização de Ciclodextrinas Na Formação de Complexos de Inclusão de Interesse Farmacêutico. Rev. De Cienc. Farm. Basica E Apl..

[28] Han X., Zhang Z., Shen H., Zheng J., Zhang G. (2019). Comparison of Structures, Physicochemical Properties and in Vitro Bioactivity between Ferulic Acid-β-Cyclodextrin Conjugate and the Corresponding Inclusion Complex. Food Res. Int..

[29] Han X., Wei T., Jiang H., Li W., Zhang G. (2020). Enhanced Water Solubility, Stability, and in Vitro Antitumor Activity of Ferulic Acid by Chemical Conjugation with Amino- b-Cyclodextrins. J. Mater. Sci..

[30] García-Padial M., Martínez-Ohárriz M.C., Navarro-Blasco I., Zornoza A. (2013). The Role of Cyclodextrins in ORAC-Fluorescence Assays. Antioxidant Capacity of Tyrosol and Caffeic Acid with Hydroxypropyl-β-Cyclodextrin. J. Agric. Food Chem..

[31] Irwin P.L., Pfeffer P.E., Doner L.W., Sapers G.M., Brewster J.D., Nagahashi G., Hicks K.B. (1994). Binding Geometry, Stoichiometry, and Thermodynamics of Cyclomalto-Oligosaccharide (Cyclodextrin) Inclusion Complex Formation with Chlorogenic Acid, the Major Substrate of Apple Polyphenol Oxidase. Carbohydr. Res..

[32] Rezaei A., Varshosaz J., Fesharaki M., Farhang A., Jafari S.M. (2019). Improving the Solubility and in Vitro Cytotoxicity (Anticancer Activity) of Ferulic Acid by Loading It into Cyclodextrin Nanosponges. Int. J. Nanomed..

[33] Kfoury M., Landy D., Auezova L., Greige-Gerges H., Fourmentin S. (2014). Effect of Cyclodextrin Complexation on Phenylpropanoids’ Solubility and Antioxidant Activity. Beilstein J. Org. Chem..

[34] Kfoury M., Lounès-Hadj Sahraoui A., Bourdon N., Laruelle F., Fontaine J., Auezova L., Greige-Gerges H., Fourmentin S. (2016). Solubility, Photostability and Antifungal Activity of Phenylpropanoids Encapsulated in Cyclodextrins. Food Chem..

[35] Shiozawa R., Inoue Y., Murata I., Kanamoto I. (2018). Effect of Antioxidant Activity of Caffeic Acid with Cyclodextrins Using Ground Mixture Method. Asian J. Pharm. Sci..

[36] Kfoury M., Geagea C., Ruellan S., Greige-Gerges H., Fourmentin S. (2019). Effect of Cyclodextrin and Cosolvent on the Solubility and Antioxidant Activity of Caffeic Acid. Food Chem..

[37] Nakhle L., Kfoury M., Greige-Gerges H., Fourmentin S. (2020). Effect of Dimethylsulfoxide, Ethanol, α- and β-Cyclodextrins and Their Association on the Solubility of Natural Bioactive Compounds. J. Mol. Liq..

[38] Rajendiran N., Mohandoss T., Thulasidhasan J. (2017). Photophysics of Caffeic, Ferulic and Sinapic Acids with α- and β-Cyclodextrins: Spectral and Molecular Modeling Studies. Int. Lett. Chem. Phys. Astron..

[39] Divakar S., Maheswaran M.M. (1997). Structural Studies on Inclusion Compounds of β-Cyclodextrin with Some Substituted Phenols. J. Incl. Phenom. Mol. Recognit. Chem..

[40] Rodrigues E., Vaz S., Gil V.M.S.S., Madalena Caldeira M., Moreira Da Silva A.M.G. (2002). Inclusion of Polyphenol Oxidase Substrates in β-Cyclodextrin: A 1H-NMR Study. J. Incl. Phenom..

[41] Chao J.-B., Tong H.B., Li Y.F., Zhang L.W., Zhang B.T. (2008). Investigation on the Inclusion Behavior of Caffeic Acid with Cyclodextrin. Supramol. Chem..

[42] Górnas P., Neunert G., Baczyński K., Polewski K. (2009). Beta-Cyclodextrin Complexes with Chlorogenic and Caffeic Acids from Coffee Brew: Spectroscopic, Thermodynamic and Molecular Modelling Study. Food Chem..

[43] Kalogeropoulos N., Konteles S., Mourtzinos I., Troullidou E., Chiou A., Karathanos V.T. (2009). Encapsulation of Complex Extracts in β-Cyclodextrin: An Application to Propolis Ethanolic Extract. J. Microencapsul..

[44] Zhang M., Li J., Zhang L., Chao J. (2009). Preparation and Spectral Investigation of Inclusion Complex of Caffeic Acid with Hydroxypropyl-β-Cyclodextrin. Spectrochim. Acta A Mol. Biomol. Spectrosc..

[45] Pinho E., Soares G., Henriques M. (2015). Evaluation of Antibacterial Activity of Caffeic Acid Encapsulated by β-Cyclodextrins. J. Microencapsul..

[46] Liu B., Zeng J., Chen C., Liu Y., Ma H., Mo H., Liang G. (2016). Interaction of Cinnamic Acid Derivatives with β-Cyclodextrin in Water: Experimental and Molecular Modeling Studies. Food Chem..

[47] Malapert A., Tomao V., Margier M., Nowicki M., Gleize B., Dangles O., Reboul E. (2018). Β-Cyclodextrin Does Not Alter the Bioaccessibility and the Uptake By Caco-2 Cells of Olive By-Product Phenolic Compounds. Nutrients.

[48] Aree T. (2019). Understanding Structures and Thermodynamics of β-Cyclodextrin Encapsulation of Chlorogenic, Caffeic and Quinic Acids: Implications for Enriching Antioxidant Capacity and Masking Bitterness in Coffee. Food Chem..

[49] Simsek T., Simsek S., Mayer C., Rasulev B. (2019). Combined Computational and Experimental Study on the Inclusion Complexes of β-Cyclodextrin with Selected Food Phenolic Compounds. Struct. Chem..

[50] Rocha B.A., Rodrigues M.R., Bueno P.C.P., De Mello Costa-Machado A.R., De Oliveira Lima Leite Vaz M.M., Nascimento A.P., Barud H.S., Berretta-Silva A.A. (2012). Preparation and Thermal Characterization of Inclusion Complex of Brazilian Green Propolis and Hydroxypropyl-β-Cyclodextrin: Increased Water Solubility of the Chemical Constituents and Antioxidant Activity. J. Therm. Anal. Calorim..

[51] Im N.R., Kim K.M., Young S.J., Park S.N. (2016). Physical Characteristics and in Vitro Skin Permeation of Elastic Liposomes Loaded with Caffeic Acid-Hydroxypropyl-β-Cyclodextrin. Korean J. Chem. Eng..

[52] Andreadelis I., Chatziathanasiadou Μ.V., Ntountaniotis D., Valsami G., Papaemmanouil C., Christodoulou E., Mitropoulou G., Kourkoutas Y., Tzakos A.G., Mavromoustakos T. (2020). Charting the Structural and Thermodynamic Determinants in Phenolic Acid Natural Product—Cyclodextrin Encapsulations. J. Biomol. Struct. Dyn..

[53] Xiong X., Zhao X., Song Z. (2014). Exploring Host-Guest Interactions of Sulfobutylether-β-Cyclodextrin and Phenolic Acids by Chemiluminescence and Site-Directed Molecular Docking. Anal. Biochem..

[54] Hunt L.E., Bourne S.A., Caira M.R. (2021). Inclusion of Hydroxycinnamic Acids in Methylated Cyclodextrins: Host-Guest Interactions and Effects on Guest Thermal Stability. Biomolecules.

[55] Zhao W., Chao J., Du R., Huang S. (2011). Spectroscopic Studies on the Inclusion Behavior between Caffenic Acid and γ-Cyclodextrin. J. Incl. Phenom. Macrocycl. Chem..

[56] Inoue Y., Suzuki K., Ezawa T., Murata I., Yokota M., Tokudome Y., Kanamoto I. (2015). Examination of the Physicochemical Properties of Caffeic Acid Complexed with γ-Cyclodextrin. J. Incl. Phenom. Macrocycl. Chem..

[57] García-Padial M., Martínez-Ohárriz M.C., Isasi J.R., Zornoza A. (2017). Sorption and Release of Natural Phenolic Antioxidants in Different Cyclodextrin Polymers. J. Agric. Food Chem..

[58] Navarro-Orcajada S., Matencio A., Vicente-Herrero C., García-Carmona F., López-Nicolás J.M. (2021). Study of the Fluorescence and Interaction between Cyclodextrins and Neochlorogenic Acid, in Comparison with Chlorogenic Acid. Sci. Rep..

[59] Aree T. (2019). Inclusion Complex of β-Cyclodextrin with Coffee Chlorogenic Acid: New Insights from a Combined Crystallographic and Theoretical Study. Acta Crystallogr. C Struct. Chem..

[60] Irwin P., Brouillette J., Giampa A., Hicks K., Gehring A., Tu S.I. (1999). Cyclomaltoheptaose (β-Cyclodextrin) Inclusion Complex Formation with Chlorogenic Acid: Hydration Enthalpy, the Solvent Entropy (Hydrophobic) Effect, and Enthalpy-Entropy Compensation. Carbohydr. Res..

[61] Alvarez-Parrilla E., De La Rosa L.A., Torres-Rivas F., Rodrigo-Garcia J., González-Aguilar G.A. (2005). Complexation of Apple Antioxidants: Chlorogenic Acid, Quercetin and Rutin by β-Cyclodextrin (β-CD). J. Incl. Phenom..

[62] Alvarez-Parrilla E., de la Rosa L.A., Rodrigo-García J., Escobedo-González R., Mercado-Mercado G., Moyers-Montoya E., Vázquez-Flores A., González-Aguilar G.A. (2007). Dual Effect of β-Cyclodextrin (β-CD) on the Inhibition of Apple Polyphenol Oxidase by 4-Hexylresorcinol (HR) and Methyl Jasmonate (MJ). Food Chem..

[63] Zhao M., Wang H., Yang B., Tao H. (2010). Identification of Cyclodextrin Inclusion Complex of Chlorogenic Acid and Its Antimicrobial Activity. Food Chem..

[64] Álvarez-Parrilla E., Palos R., De La Rosa L.A., Frontana-Uribe B.A., González-Aguilar G.A., Machi L., Ayala-Zavala J.F. (2010). Formation of Two 1:1 Chlorogenic Acid: β-Cyclodextrin Complexes at PH 5: Spectroscopic, Thermodynamic and Voltammetric Study. J. Mex. Chem. Soc..

[65] Budryn G., Nebesny E., Pałecz B., Rachwał-Rosiak D., Hodurek P., Miśkiewicz K., Oracz J., Zyzelewicz D. (2014). Inclusion Complexes of β-Cyclodextrin with Chlorogenic Acids (CHAs) from Crude and Purified Aqueous Extracts of Green Robusta Coffee Beans (*Coffea canephora* L.). Food Res. Int..

[66] Shao P., Zhang J., Fang Z., Sun P. (2014). Complexing of Chlorogenic Acid with β-Cyclodextrins: Inclusion Effects, Antioxidative Properties and Potential Application in Grape Juice. Food Hydrocoll..

[67] Budryn G., Zaczyńska D., Rachwał-Rosiak D. (2016). Changes of Free and Nanoencapsulated Hydroxycinnamic Acids from Green Coffee Added to Different Food Products during Processing and in Vitro Enzymatic Digestion. Food Res. Int..

[68] Budryn G., Zaczyńska D., Żyżelewicz D., Grzelczyk J., Zduńczyk Z., Juśkiewicz J. (2017). Influence of the Form of Administration of Chlorogenic Acids on Oxidative Stress Induced by High Fat Diet in Rats. Plant. Foods Hum. Nutr..

[69] Chao J., Wang H., Zhao W., Zhang M., Zhang L. (2012). Investigation of the Inclusion Behavior of Chlorogenic Acid with Hydroxypropyl-β-Cyclodextrin. Int. J. Biol. Macromol..

[70] Uekama K., Otagiri M., Kanie Y., Tanaka S., Ikeda K. (1975). Inclusion Complexes of Cinnamic Acids with Cyclodextrins. Mode of Inclusion in Aqueous Solution. Chem. Pharm. Bull..

[71] Krajl B., Smidovnik A., Kobe J. (2009). Mass Spectrometric Investigations of α- and β- Cyclodextrin Complexes with Ortho-, Meta- and Para- Coumaric Acids by Negative Mode Electrospray Ionization. Rapid Commun. Mass Spectrom..

[72] Stražišar M., Andrenšek S., Šmidovnik A. (2008). Effect of β-Cyclodextrin on Antioxidant Activity of Coumaric Acids. Food Chem..

[73] Kim J.-S. (2020). Synthesis and Characterization of Phenolic Acid/Hydroxypropyl-β-Cyclodextrin Inclusion Complexes. Prev. Nutr. Food Sci..

[74] Qi A.-D., Li L., Liu Y. (2003). Molecular Binding Ability and Selectivity of Natural α-, β-, γ-Cyclodextrins and Oligo (Ethylenediamino) Modified β-Cyclodextrins with Chinese Traditional Medicines. J. Incl. Phenom..

[75] Anselmi C., Centini M., Maggiore M., Gaggelli N., Andreassi M., Buonocore A., Beretta G., Facino R.M. (2008). Non-Covalent Inclusion of Ferulic Acid with α-Cyclodextrin Improves Photo-Stability and Delivery: NMR and Modeling Studies. J. Pharm. Biomed. Anal..

[76] Monti D., Tampucci S., Chetoni P., Burgalassi S., Saino V., Centini M., Staltari L., Anselmi C. (2011). Permeation and Distribution of Ferulic Acid and Its α-Cyclodextrin Complex from Different Formulations in Hairless Rat Skin. AAPS PharmSciTech.

[77] González-Mondragón E., Torralba-González A., García-Gutiérrez P., Robles-González V.S., Salazar-Govea A.Y., Zubillaga R.A. (2016). Thermodynamic Analysis of Ferulate Complexation with α-, β- And γ-Cyclodextrins. Acta.

[78] Mori T., Tsuchiya R., Doi M., Nagatani N., Tanaka T. (2019). Solubilization of Ultraviolet Absorbers by Cyclodextrin and Their Potential Application in Cosmetics. J. Incl. Phenom. Macrocycl. Chem..

[79] Zhang M., Li J., Jia W., Chao J., Zhang L. (2009). Theoretical and Experimental Study of the Inclusion Complexes of Ferulic Acid with Cyclodextrins. Supramol. Chem..

[80] Li Y., Yu H., Cai Y., Yuan C., Chen S., Ding T., Liu D., Hu Y. (2020). Ferulic Acid-β-Cyclodextrin Inclusion Complexes: Application on the Preservation of Hairtail (*Trichiurus lepturus*). Int. J. Food Prop..

[81] Wang J., Cao Y., Sun B., Wang C. (2011). Characterisation of Inclusion Complex of Trans-Ferulic Acid and Hydroxypropyl-β-Cyclodextrin. Food Chem..

[82] Olga G., Styliani C., Ioannis R.G. (2015). Coencapsulation of Ferulic and Gallic Acid in Hp-b-Cyclodextrin. Food Chem..

[83] Hsu C.M., Yu S.C., Tsai F.J., Tsai Y. (2019). Characterization of in Vitro and in Vivo Bioactivity of a Ferulic Acid-2-Hydroxypropyl-β-Cyclodextrin Inclusion Complex. Colloids Surf. B Biointerfaces.

[84] Celebioglu A., Uyar T. (2020). Development of Ferulic Acid /Cyclodextrin Inclusion Complex Nano Fi Bers for Fast-Dissolving Drug Delivery System. Int. J. Pharm..

[85] Casolaro M., Anselmi C., Picciocchi G. (2005). The Protonation Thermodynamics of Ferulic Acid/γ-Cyclodextrin Inclusion Compounds. Thermochim. Acta.

[86] Çelik S.E., Özyürek M., Tufan A.N., Güçü K., Apak R. (2011). Spectroscopic Study and Antioxidant Properties of the Inclusion Complexes of Rosmarinic Acid with Natural and Derivative Cyclodextrins. Spectrochim. Acta A Mol. Biomol. Spectrosc..

[87] Medronho B., Valente A.J.M., Costa P., Romano A. (2014). Inclusion Complexes of Rosmarinic Acid and Cyclodextrins: Stoichiometry, Association Constants, and Antioxidant Potential. Colloid Polym. Sci..

[88] Aksamija A., Polidori A., Plasson R., Dangles O., Tomao V. (2016). The Inclusion Complex of Rosmarinic Acid into Beta-Cyclodextrin: A Thermodynamic and Structural Analysis by NMR and Capillary Electrophoresis. Food Chem..

[89] Fateminasab F., Bordbar A.K., Shityakov S., Saboury A.A. (2020). Molecular Insights into Inclusion Complex Formation between β- and γ-Cyclodextrins and Rosmarinic Acid. J. Mol. Liq..

[90] Veras K.S., Silveira Fachel F.N., Delagustin M.G., Teixeira H.F., Barcellos T., Henriques A.T., Bassani V.L., Koester L.S. (2019). Complexation of Rosmarinic Acid with Hydroxypropyl-β-Cyclodextrin and Methyl-β-Cyclodextrin: Formation of 2:1 Complexes with Improved Antioxidant Activity. J. Mol. Struct..

[91] Jambhekar S.S., Breen P. (2016). Cyclodextrins in Pharmaceutical Formulations II: Solubilization, Binding Constant, and Complexation Efficiency. Drug Discov. Today.

[92] Cirri M., Maestrelli F., Orlandini S., Furlanetto S., Pinzauti S., Mura P. (2005). Determination of Stability Constant Values of Flurbiprofen-Cyclodextrin Complexes Using Different Techniques. J. Pharm. Biomed. Anal..

[93] Singh R., Tønnesen H.H., Vogensen S.B., Loftsson T., Másson M. (2010). Studies of Curcumin and Curcuminoids. XXXVI. The Stoichiometry and Complexation Constants of Cyclodextrin Complexes as Determined by the Phase-Solubility Method and UV-Vis Titration. J. Incl. Phenom. Macrocycl. Chem..

[94] Chadha R., Kashid N., Saini A. (2004). Account of Analytical Techniques Employed for the Determination of Thermodynamics of Inclusion Complexation of Drugs with Cyclodextrins. J. Sci. Ind. Res..

[95] Mitra A., Seaton P.J., Capitani J.F., Assarpour A. (1998). Unprecedented Concentration Dependent Chemical Shift Variation in 1H NMR Studies: A Problem in Structure Elucidation and the Study of Molecular Recognition. J. Indian Chem. Soc..

[96] Hoenigman S.M., Evans C.E. (1996). Improved Accuracy and Precision in the Determination of Association Constants. Anal. Chem..

[97] Schönbeck C., Westh P., Madsen J.C., Larsen K.L., Städe L.W., Holm R. (2010). Hydroxypropyl-Substituted β-Cyclodextrins: Influence of Degree of Substitution on the Thermodynamics of Complexation with Tauroconjugated and Glycoconjugated Bile Salts. Langmuir.

[98] Loukas Y.L. (1997). Evaluation of the Methods for the Determination of the Stability Constant of Cyclodextrin-Chlorambucil Inclusion Complexes. J. Pharm. Biomed. Anal..

[99] Loftsson T., Brewster M.E. (1996). Pharmaceutical Applications of Cyclodextrins. 1. Drug Solubilization and Stabilization. J. Pharm. Sci..

[100] Rekharsky M.V., Inoue Y. (1998). Complexation Thermodynamics of Cyclodextrins. Chem. Rev..

[101] Loftsson T., Brewster M.E. (2012). Cyclodextrins as Functional Excipients: Methods to Enhance Complexation Efficiency. J. Pharm. Sci..

[102] Loftsson T., Hreinsdóttir D., Másson M. (2007). The Complexation Efficiency. J. Incl. Phenom. Macrocycl. Chem..

[103] Anselmi C., Centini M., Ricci M., Buonocore A., Granata P., Tsuno T., Facino R.M. (2006). Analytical Characterization of a Ferulic Acid/γ-Cyclodextrin Inclusion Complex. J. Pharm. Biomed. Anal..

[104] Salústio P.J., Pontes P., Conduto C., Sanches I., Carvalho C., Arrais J., Marques H.M.C. (2011). Advanced Technologies for Oral Controlled Release: Cyclodextrins for Oral Controlled Release. AAPS PharmSciTech.

[105] Ahmad I., Ahmed S., Anwar Z., Sheraz M.A., Sikorski M. (2016). Photostability and Photostabilization of Drugs and Drug Products. Int. J. Photoenergy.

[106] Sovizi M.R. (2010). Thermal Behavior of Drugs: Investigation on Decomposition Kinetic of Naproxen and Celecoxib. J. Therm. Anal. Calorim..

[107] Razboršek M.I. (2011). Stability Studies on Trans-Rosmarinic Acid and GC-MS Analysis of Its Degradation Product. J. Pharm. Biomed. Anal..

[108] Mäkilä L., Laaksonen O., Alanne A.L., Kortesniemi M., Kallio H., Yang B. (2016). Stability of Hydroxycinnamic Acid Derivatives, Flavonol Glycosides, and Anthocyanins in Black Currant Juice. J. Agric. Food Chem..

[109] Santos N.A., Cordeiro A.M.T.M., Damasceno S.S., Aguiar R.T., Rosenhaim R., Carvalho Filho J.R., Santos I.M.G., Maia A.S., Souza A.G. (2012). Commercial Antioxidants and Thermal Stability Evaluations. Fuel.

[110] Mota F.L., Queimada A.J., Pinho S.P., Macedo E.A. (2008). Aqueous Solubility of Some Natural Phenolic Compounds. Ind. Eng. Chem. Res..

